# A systematic literature review and (network) meta-analysis of the effectiveness of ceftolozane/tazobactam versus aminoglycosides/polymyxins and ceftazidime/avibactam for treating adult patients with multidrug-resistant *Pseudomonas aeruginosa* infections

**DOI:** 10.1128/aac.00735-25

**Published:** 2026-01-14

**Authors:** Hannah Collings, Medi Stone, Maria Chrysostomou, Alex Hirst, Todd Edward Waldenberg, Emre Yucel, Thomas Iodise

**Affiliations:** 1 Adelphi Values PROVE, Bollington, United Kingdom; 2 Merck & Co., Inc 2793 , Rahway, New Jersey, USA; University of Pennsylvania Perelman School of Medicine, Philadelphia, Pennsylvania, USA

**Keywords:** MDR-*P. aeruginosa*, ceftolozane/tazobactam, C/T, ceftazidime/avibactam, aminoglycosides/polymyxins, network meta-analysis

## Abstract

Multidrug-resistantant (MDR) *Pseudomonas aeruginosa* is a major public health concern necessitating new antimicrobials. There are new antimicrobials available with activity against MDR-*P. aeruginosa,* but there is a lack of robust evidence synthesis to guide clinical decision-making for patients with infections caused by MDR-*P. aeruginosa*. This study, which was supported by Merck & Co., Inc., aimed to evaluate the real-world effectiveness of ceftolozane/tazobactam (C/T) compared to other commonly used therapies (aminoglycosides/polymyxins and ceftazidime/avibactam [CZA]) among adults with MDR-*P. aeruginosa* infections. A systematic literature review was conducted to identify real-world clinical and healthcare-resource utilization outcomes for C/T versus comparators. A feasibility assessment excluded comparators that were not aminoglycosides/polymyxins or CZA due to insufficient data for comparisons. A meta-analysis and network meta-analysis (NMA) were conducted on the included studies. Heterogeneity between the studies was calculated using *I*^2^ statistic. The NMA displayed statistically significant results for clinical cure (odds ratio [OR] = 0.308, 95% CI = 0.168–0.515) and all-cause mortality (OR = 1.651, 95% CI = 1.114–2.501) for C/T versus aminoglycosides/polymyxins, while microbiological cure and length of stay did not display statistical significance. However, comparisons for C/T versus CZA demonstrated no statistical significance for any of the outcomes explored. These findings suggest that C/T is more likely to achieve clinical cure and less likely to result in all-cause mortality compared to aminoglycosides/polymyxins. In the absence of head-to-head trials, this real-world evidence indicates potential advantages of using C/T over aminoglycosides/polymyxins for MDR-*P. aeruginosa* infections. Larger prospective studies with standardized outcome measures are needed to further inform clinical decision-making.

## INTRODUCTION

*Pseudomonas aeruginosa* is a frequent cause of hospital-acquired infections, including hospital-acquired bacterial pneumonia (HABP), ventilator-associated bacterial pneumonia (VABP), complicated urinary tract infections (cUTIs), and complicated intra-abdominal infections (cIAIs) (1–4). The emergence of multidrug-resistant (MDR) *P. aeruginosa* has exemplified the burden of infections caused by *P. aeruginosa*, as demonstrated by the mortality rate for MDR-*P. aeruginosa* infections (44.6%) being almost double the mortality rate for non-MDR-*P. aeruginosa* infections (24.8%) (5). As a testament to the seriousness of MDR-*P. aeruginosa* infections worldwide, the World Health Organization has classified MDR-*P. aeruginosa* as a critical pathogen, where there is a substantial need for new therapies to counteract this imminent public health crisis (6).

The rise of MDR-*P. aeruginosa* has resulted in the reduced effectiveness of traditional anti-pseudomonal β-lactams (such as meropenem, piperacillin/tazobactam, ceftazidime, or cefepime) when used as an empiric monotherapy for MDR-*P. aeruginosa* infections (7). Historically, polymyxins and aminoglycosides were used as last-line agents to treat MDR Gram-negative infections (8). However, the increase in antimicrobial resistance (AMR) has led to the renewed use of polymyxins and aminoglycosides in clinical practice, often in combination with conventional anti-pseudomonal β-lactams with limited or no *in vitro* activity against most clinical MDR-*P. aeruginosa* isolates. Novel therapies such as ceftolozane/tazobactam (C/T) and ceftazidime/avibactam (CZA) with high *in vitro* microbiologic activity against MDR-*P. aeruginosa* and improved safety profiles relative to polymyxins and aminoglycosides are now available (9–12). The availability of these novel therapies has relegated polymyxin and aminoglycoside-combination regimens to second-line agents across several recent expert guidance documents (13).

Despite recent expert recommendations, there remains considerable variability in prescribing practices for the treatment of patients with highly resistant Gram-negative infections. For example, first-line treatments for *P. aeruginosa* infections may include β-lactam/β-lactamase-inhibitor combinations such as piperacillin-tazobactam or cephalosporins such as cefepime or fluoroquinolones like levofloxacin (14). The variability in prescribing practices is partly due to the approval of new agents by regulatory authorities being largely based on non-inferiority (NI) randomized control trials (RCTs) (15–18). Along with the well-known challenges in designing and interpreting NI-RCTs (19, 20), most NI-RCTs involving serious Gram-negative infections exclude patients with suspected or documented infections caused by pathogens resistant to the active comparator, in order to maintain clinical equipoise (15–18, 21). Consequently, there is limited evidence for the efficacy of novel therapies against pathogens like MDR-*P. aeruginosa* (22). This has significant implications for clinical practice, as data from NI-RCTs do not offer clinicians enough information to determine the best therapies for treating highly resistant Gram-negative infections, such as MDR-*P. aeruginosa,* and there are not sufficient data to conduct robust NI-RCT-based meta-analyses (MAs) or network meta-analyses (NMAs) (23, 24).

Several real-world comparative effectiveness studies have been conducted to better understand the outcomes associated with available therapies for treating patients with MDR-*P. aeruginosa* infections (9–11, 25). While these studies have contributed valuable insights to treatment decisions, there is significant variability in study populations, treatment comparators, and endpoints. Additionally, most of these studies are limited by small sample sizes and were not powered to detect clinically meaningful differences in primary outcomes between treatments. Given these limitations, there is a clear need for a well-designed evidence synthesis to guide clinical decisions for patients with serious infections caused by highly resistant Gram-negative pathogens like MDR-*P. aeruginosa*.

The primary goal of this research, which was supported by Merck & Co., Inc., was to evaluate the real-world effectiveness of C/T compared with other commonly used therapies (i.e., aminoglycoside-containing regimens, polymyxin-containing regimens, and CZA) among adult patients with MDR-*P. aeruginosa* infections. A systematic literature review (SLR) captured real-world effectiveness studies which assessed clinical and healthcare-resource utilization outcomes in adult patients with MDR-*P. aeruginosa* infections treated with C/T, CZA, aminoglycosides/polymyxins, and other comparators (see Table S1). Publications that reported on the following widely reported outcomes were prioritized: clinical cure, microbiological cure/failure, all-cause mortality, and length of stay (LOS). A feasibility assessment excluded any publication that included comparators that were not aminoglycosides, polymyxins, or CZA. Following the feasibility assessment, an MA was conducted including C/T data from both single-arm and multi-arm trials to identify a single pooled estimate with 95% CIs across studies for all outcomes. Additionally, an NMA was then conducted using both common fixed effects (FE) and random effects (RE) models to allow ranking of treatments in terms of efficacy. Heterogeneity between the studies was calculated using *I*^2^ statistic. In the absence of RCTs, this approach allowed real-world comparisons of C/T versus aminoglycosides/polymyxins and CZA to help inform to aid clinical decision-making for patients with cUTIs, cIAIs, and HABP/VABP.

## RESULTS

### Included publications

**Fig 1:**
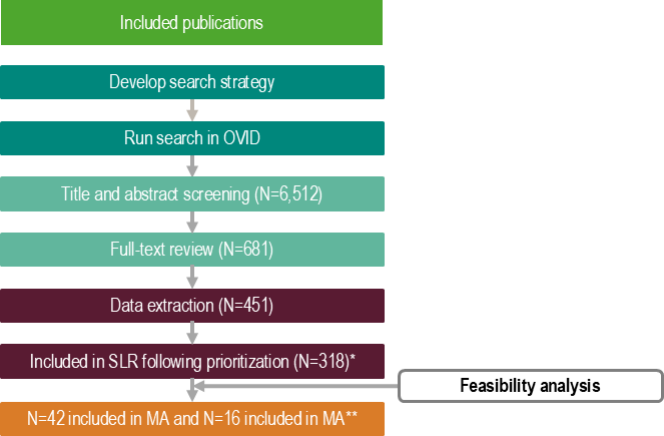
SLR Preferred Reporting Items for Systematic Reviews and Meta-Analyses (PRISMA) diagram. *The SLR prioritization excluded publications that were case studies or included <10 patients, publications that reported on populations that were not exclusively infected with *P. aeruginosa* and publications with homogenous clinical outcomes. **Feasibility analysis excluded any publication that included comparators that were not: aminoglycosides, polymyxins, or CZA. Confidential-not for public consumption or distribution.

The SLR searches identified 6,512 records which were screened according to title/abstract and subsequently according to the full text. The SLR identified 451 publications which met the Population, Intervention, Comparator, Outcome, Time, Study design (PICOTS) criteria and were extracted into the data extraction form (DEF) (Fig. 1). Prioritization criteria were then applied, which excluded publications that were case studies or included <10 patients, publications that reported on populations that were not exclusively infected with *P. aeruginosa,* and publications that did not report on the following outcomes: clinical and microbiological cure, all-cause mortality, and LOS. Following the prioritization criteria, a total of 318 publications were included in the SLR.

**Fig 2:**
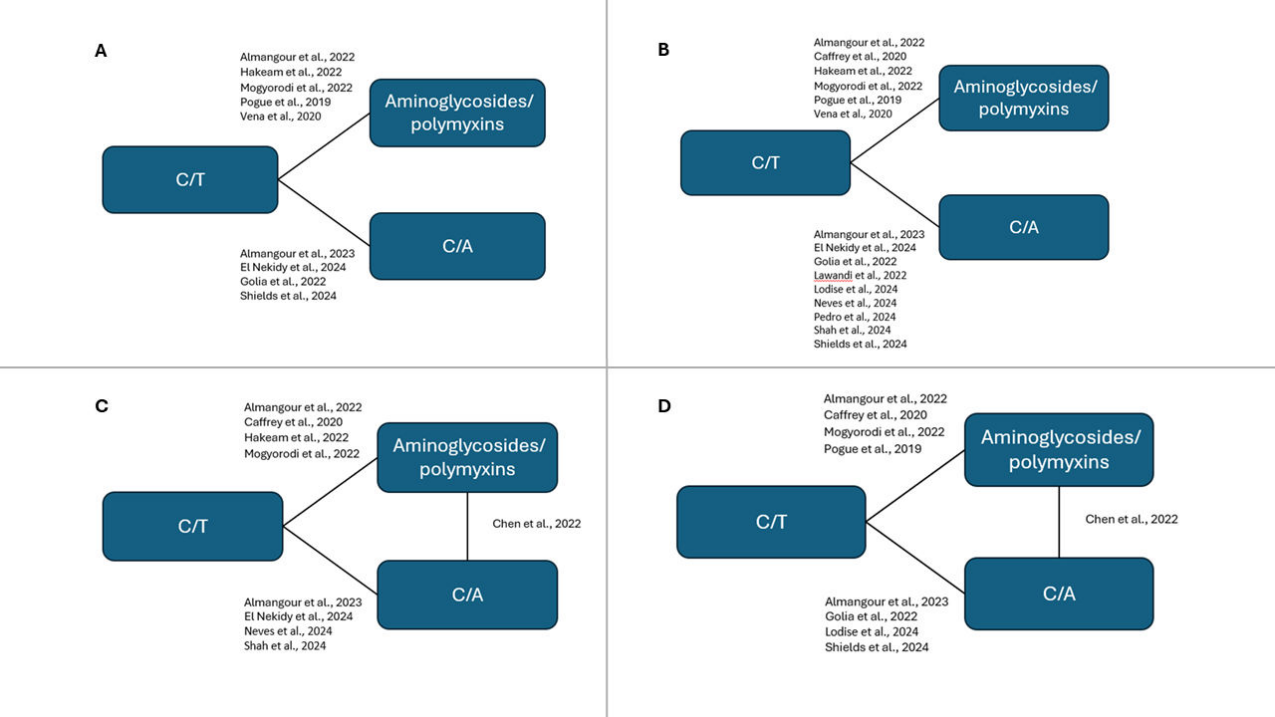
Network diagrams for outcomes: clinical cure (**A**), all-cause mortality (**B**), microbiological cure (**C**), and LOS (**D**).

**Fig 3:**
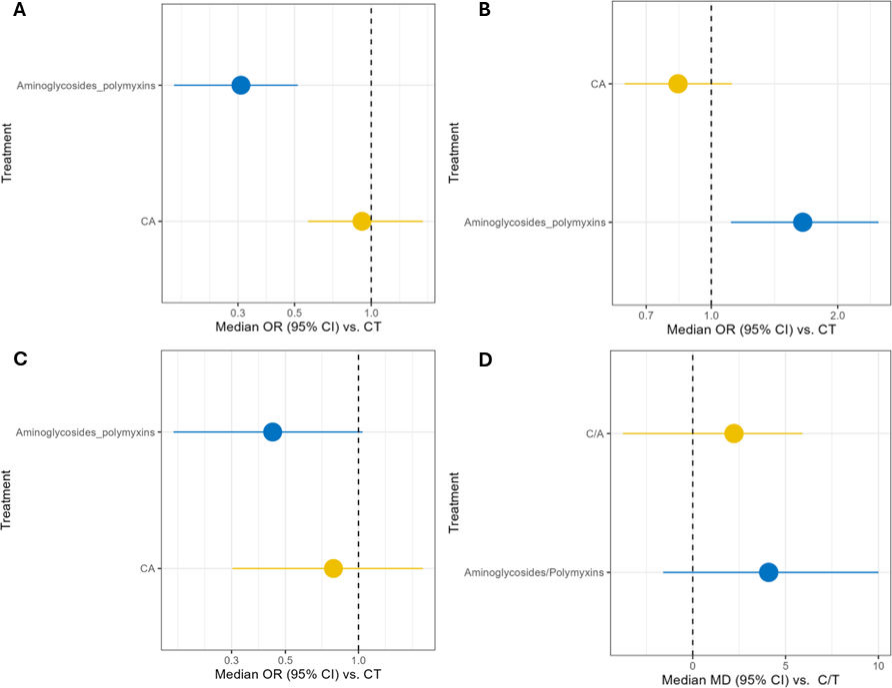
The REs network meta-analysis forest plots for (**A**) clinical cure, (**B**) all-cause mortality, (**C**) microbiological cure, and (**D**) LOS.

Of the 318 publications, 42 were included in the MA and 16 in the NMA, following the feasibility assessment which excluded publications on comparators that were not CZA or aminoglycoside/polymyxins. This was due to the lack of data in publications for the other comparative treatments. Studies for each outcome comparing C/T with CZA and aminoglycosides/polymyxins for the MA are shown in Table 1 and for the NMA are shown in Table 2. The network diagrams for each outcome are presented in Fig. 2, and the RE NMA forest plots for each outcome are presented in Fig. 3.

**TABLE 1:** Included studies in meta-analysis and associated included outcomes (*N* = 42)

Citation	Clinical cure	All-cause mortality	Microbiological cure	Length of stay
Almangour et al. (26)	✓	✓	✓	✓
Almangour et al. (27)	✓	✗	✓	✓
Caffrey et al. (25)	✗	✗	✓	✓
Fernández-Cruz et al. (28)	✓	✓	✗	✓
Hakeam et al. (29)	✓	✓	✓	✗
Lawandi et al. (30)	✗	✗	✗	✗
Golia et al. (31)	✓	✗	✗	✓
Mills et al. (32)	✓	✗	✗	✓
Mogyoródi et al. (33)	✓	✓	✓	✓
Vena et al. (11)	✓	✓	✗	✗
Pogue et al. (9)	✓	✗	✗	✓
Shah et al. (34)	✗	✓	✓	✗
Shields et al. (35)	✓	✓	✗	✓
Mendes Pedro et al. (36)	✗	✓	✗	✗
Neves et al. (37)	✗	✗	✓	✗
Shields et al. (38)	✓	✓	✗	✓
Vance et al. (39)	✓	✗	✗	✗
El Nekidy et al. (40)	✓	✓	✓	✗
Lodise et al. (41)	✗	✓	✗	✓
Balandin et al. (42)	✓	✓	✓	✓
Bassetti et al. (43)	✓	✓	✗	✗
Margarita Beltran-Garcia et al. (44)	✓	✓	✗	✗
Bosaeed et al. (45)	✓	✓	✓	✗
Castón et al. (46)	✓	✓	✓	✗
Diaz-Cañestro et al. (47)	✓	✓	✗	✗
Dinh et al. (48)	✓	✓	✓	✓
Elabor et al. (49)	✓	✓	✓	✗
Escolà-Vergé et al. (50)	✓	✓	✓	✗
Gallagher et al. (51)	✓	✓	✓	✓
Gerlach et al. (52)	✓	✓	✓	✓
Gioia et al. (53)	✓	✓	✓	✗
Gudiol et al. (54)	✗	✓	✗	✗
Haidar et al. (55)	✓	✓	✗	✗
Hart et al. (56)	✓	✓	✗	✓
Hart et al. (57)	✓	✓	✗	✓
Henry et al. (58)	✓	✓	✗	✗
Jorgensen et al. (59)	✓	✓	✗	✗
Levy-Bachelot et al. (60)	✓	✓	✗	✗
Munita et al. (61)	✓	✓	✓	✗
Navarrete-Rouco et al. (62)	✓	✓	✗	✗
Rodríguez-Núñez et al. (63)	✓	✓	✓	✗
Xipell et al. (64)	✓	✓	✓	✗
Total	35	34	20	17

**TABLE 2:** Included studies in network meta-analysis and associated included outcomes (*N* = 16)

Citation	Clinical cure	All-cause mortality	Microbiological cure	Length of stay
Almangour et al. (26)	✓	✓	✓	✓
Almangour et al. (27)	✓	✓	✓	✓
Caffrey et al. (25)	✓	✗	✓	✓
Hakeam et al. (29)	✗	✓	✓	✓
Lawandi et al. (30)	✗	✗	✗	✓
Golia et al. (31)	✓	✓	✗	✓
Mogyoródi et al. (33)	✓	✓	✓	✓
Vena et al. (11)	✗	✓	✗	✓
Pogue et al. (9)	✓	✓	✗	✓
Shah et al. (34)	✗	✗	✓	✓
Mendes Pedro et al. (36)	✗	✗	✗	✓
Neves et al. (37)	✗	✗	✓	✓
El Nekidy et al. (40)	✗	✓	✓	✓
Lodise et al. (41)	✓	✗	✗	✓
Shields et al. (35)	✓	✓	✗	✓
Chen et al. (10)	✓	✗	✓	✗
Total	9	9	9	15

### Clinical cure

**Fig 4:**
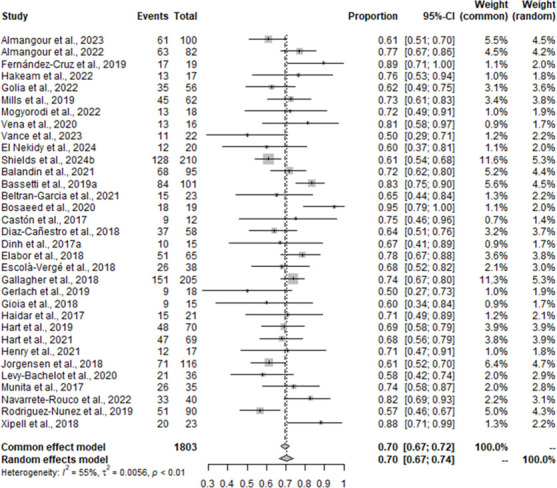
The meta-analysis forest plot for clinical cure for C/T versus comparators identifying single pooled estimates with 95% CI.

Results of the MA for clinical cure among studies that included C/T versus the pooled comparator groups (i.e., CZA and aminoglycosides/ polymyxins-containing regimens) are displayed in Fig. 4. Definitions of clinical cure were variable and included survival at 30 days after therapy completion, resolution of infection signs and symptoms, success at day 30, clinical success at day 14, and 90-day survival and clinical success. Overall, substantial heterogeneity was observed as demonstrated by an *I*^2^ statistic of 55%. Both FE and RE models were performed to add robustness to the methodological stratification. The FE model found a mean of 0.70 (95% CI = 0.67–0.72), and the RE model found a mean of 0.70 (95% CI = 0.67–0.74) (Fig. 4), suggesting that approximately 70% of patients treated with C/T achieve clinical cure across the included studies.

Results of the NMA for clinical cure are shown in Fig. 3A. The RE model had a higher Deviance Information Criterion (DIC), indicating a relative improvement in fit and therefore was selected over the FE model for the NMA. For the RE model, aminoglycosides/polymyxins demonstrated an odds ratio (OR) of 0.308 (95% CI = 0.168–0.515), suggesting that aminoglycosides/polymyxins are less likely to achieve clinical cure versus C/T, as the result was statistically significant. CZA did not display statistical significance versus C/T for clinical cure (OR = 0.920, 95% CI = 0.565–1.595). Sensitivity analyses were conducted to reduce potential bias from sources of heterogeneity by restricting studies to those that reported results for clinical cure up to 30 days (Fig. S1) and excluding studies conducted in Asia. The results for the sensitivity analysis for clinical cure within 30 days improved the results for C/T, with all point estimates being in favor of C/T and the comparison to aminoglycosides/polymyxins being statistically significant. The sensitivity analysis excluding studies in Asia showed results aligned with the base case, with the point estimates for C/T versus aminoglycosides/polymyxins and C/T versus CZA being slightly improved; however, there was no statistical significance.

### All-cause mortality

**Fig 5:**
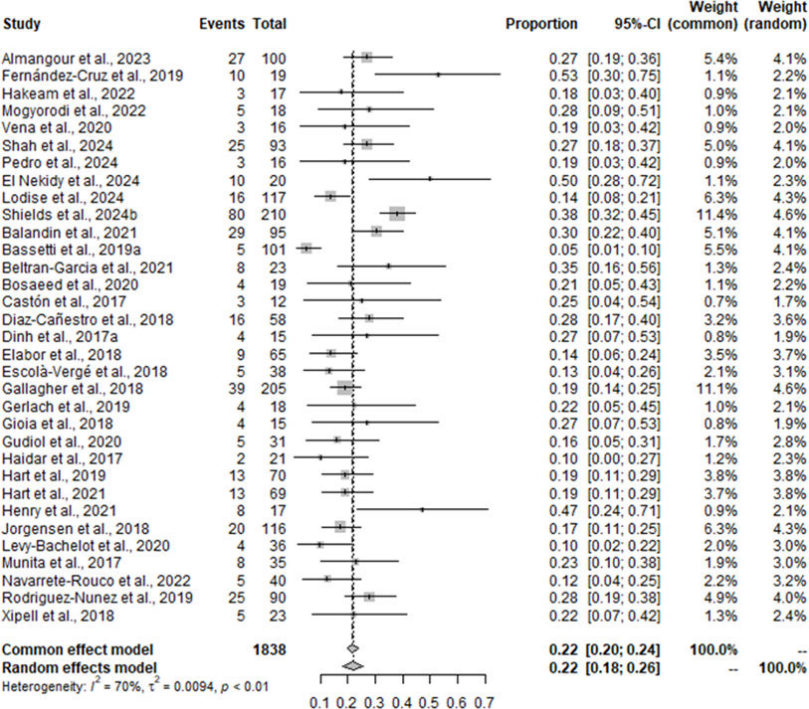
The meta-analysis forest plot for all-cause mortality for C/T versus comparators identifying single pooled estimates with 95% CI.

Results of the MA for all-cause mortality within 30 days among studies that included C/T versus the pooled comparator group (i.e., CZA and aminoglycosides/ polymyxins-containing regimens) are displayed in Fig. 5. The heterogeneity between studies reporting all-cause mortality was substantial, with an *I*^2^ score of 70%. The MA for all-cause mortality for C/T was estimated at 0.22 for both the FE (95% CI = 0.20–0.24) and RE (95% CI = 0.18–0.26) models, suggesting that 22% of patients being treated with C/T die within 30 days based on the 34 studies that assumed an all-cause mortality definition (including 14, 28, and 30-day in-hospital, ICU, infection-related, and all-cause mortality).

Results of the NMA are shown in Fig. 3B. For all-cause mortality within 30 days, the RE model was selected for the NMA due to having a higher DIC. Aminoglycosides/ polymyxins demonstrated a statistically significant OR of 1.651 (95% CI = 1.114–2.501), suggesting that this treatment results in an increased likelihood of mortality versus C/T. CZA had an OR of 0.833 (95% CI = 0.622–1.120), suggesting a lower likelihood of mortality compared to C/T; however, these results are not statistically significant (Fig. 3B). Sensitivity analyses restricted the studies to only those that reported all-cause mortality at 30 days (Supplementary material, Fig. S1) and restricted based on geographical region (studies conducted in Asia were excluded). The sensitivity analysis, restricting to studies that report 30-day all-cause mortality, identified slightly more favorable results for C/T; however, there were no changes to the statistical significance. When the analysis was restricted to studies not conducted in Asia, the results from the sensitivity analysis suggested the exclusion of studies conducted in Asia to not be a driving factor in heterogeneity.

### Microbiological cure

**Fig 6:**
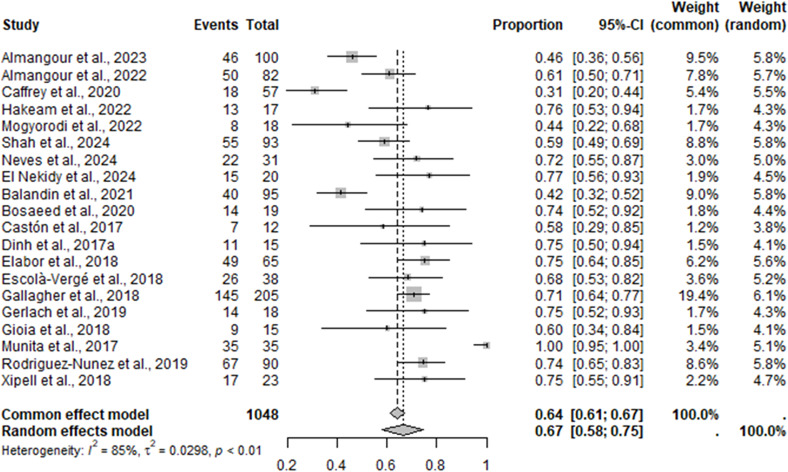
The meta-analysis forest plot for microbiological cure for C/T versus comparators identifying single pooled estimates with 95% CI.

Results of the MA for microbiologic cure are shown in Fig. 6. The definitions of microbiological cure were variable and included no growth of microbiological culture, negative follow-up culture, the absence of isolation of *P. aeruginosa*, and absence of microbiological failure. An MA to determine the microbiological cure of C/T was conducted using FE and RE models. An *I*^2^ value of 85% indicated there was a considerable amount of heterogeneity between studies. The MA presented a mean of 0.64 (95% CI = 0.61–0.67) and 0.67 (95% CI = 0.58–0.75) for the FE and RE models, respectively. These results suggest that 64%–67% of patients treated with C/T achieve microbiological cure.

Results of the NMA for microbiologic cure are shown in Fig. 3C. For the NMA, the RE model for microbiological cure was selected over the FE model due to the higher DIC. The results of the RE model were ORs of 0.443 (95% CI = 0.173–1.040) and 0.789 (95% CI = 0.303–1.842) for C/T versus aminoglycosides/polymyxins and C/T versus CZA, respectively (Fig. 3C). Sensitivity analyses (Fig. S1) were conducted to focus on studies with a definition of microbiological cure relating to eradication by 30 days and excluding studies conducted in Asia. The results from the sensitivity analyses were consistent with the primary results of the NMA for microbiologic cure. However, the number of studies was limited in both sensitivity analyses, resulting in uncertainty and suggesting the evidence base was not sufficient to conduct this analysis.

### LOS

**Fig 7:**
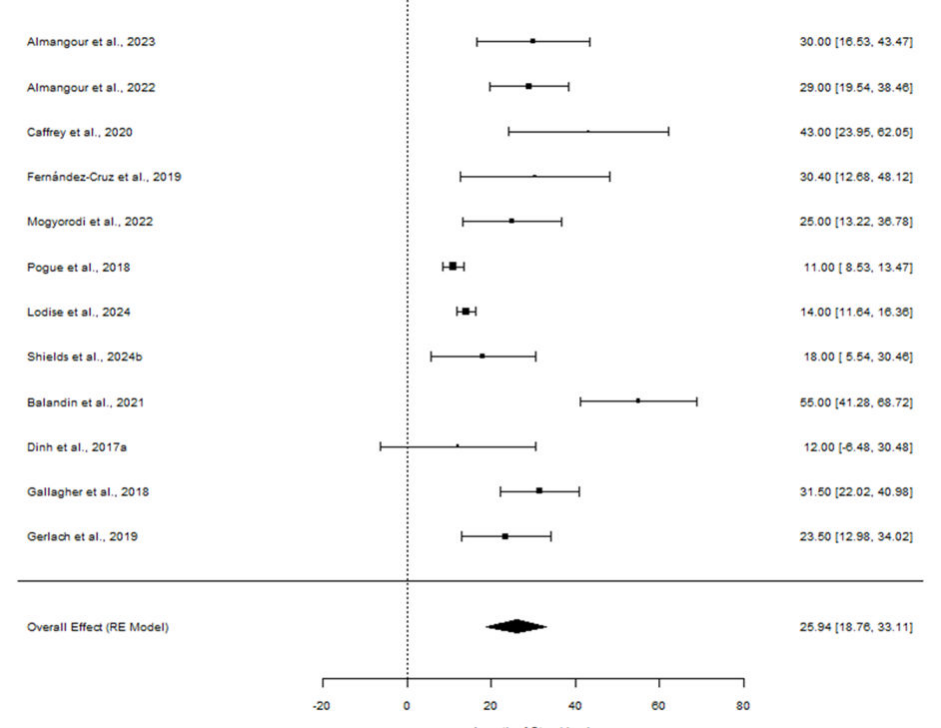
The meta-analysis forest plot for LOS for C/T versus comparators identifying the overall effect of the RE model with 95% CI.

Results of the MA for LOS are shown in Fig. 7. For the MA, the mean LOS was estimated using an RE model, as 25.94 days (95% CI = 18.76–33.11). This suggests that patients being treated with C/T stay in hospital for an average of 26 days (Fig. 7).

Results of the NMA for LOS are shown in Fig. 3D. The RE model for the continuous NMA for LOS was selected due to the higher DIC. Aminoglycosides/polymyxins had a mean difference (MD) of 4.090 (95% CI = −1.594 to 10.000); therefore, they were associated with longer LOS when compared to C/T. The MD for CZA was 2.222 (95% CI = −3.759 to 5.914) (Fig. 3D) inferring a shorter LOS compared to C/T. However, no results were statistically significant as all CIs crossed the origin. A sensitivity analysis was conducted where studies conducted in Asia were excluded. The results of the sensitivity analysis became slightly more favorable for C/T versus CZA; however, the results remain statistically non-significant.

## DISCUSSION

The comprehensive SLR was conducted to gain a deeper understanding of the outcomes associated with C/T compared to other commonly used treatments for adult patients with MDR-*P. aeruginosa* infections across available real-world evidence studies. As part of the SLR, 318 studies were identified that evaluated clinical and healthcare-resource utilization outcomes associated with available treatments for MDR-*P. aeruginosa* infections. There was considerable variability across these studies in populations, treatment comparators, and endpoints. Additionally, many studies were limited by small sample sizes and lacked the power to detect clinically meaningful differences in primary outcomes between treatments. Therefore, prioritization criteria were applied which excluded publications that were case studies or included <10 patients, reported on populations that were not exclusively infected with *P. aeruginosa* or included homogenous clinical outcomes to ensure that the included data were reliable and generalizable. A feasibility assessment excluded studies reporting comparator treatments other than CZA and aminoglycosides/polymyxins due to lack of data. Following the feasibility assessment, 42 and 16 studies were deemed suitable for the MA and NMA, respectively. The MA was conducted to identify a single pooled estimate with 95% CIs across studies for all outcomes (clinical cure, all-cause mortality, microbiological cure, and LOS). The NMA was conducted using both FE and RE models to allow ranking of treatments in terms of efficacy. Heterogeneity between the studies was calculated using *I*^2^ statistic.

Overall, several key findings emerged from this study. The results suggest that C/T may be more effective than aminoglycoside/polymyxin therapy in treating MDR-*P. aeruginosa* infections, particularly in terms of clinical cure and survival, as observed in the available real-world effectiveness studies. This is reflected in the ORs from the NMA for aminoglycoside/polymyxin treatment compared to C/T for clinical cure (OR = 0.323, 95% CI = 0.216–0.476) and all-cause mortality (OR = 1.645; 95% CI = 1.123–2.448). Aminoglycoside/polymyxin therapy was also associated with lower odds of achieving microbiologic cure compared to C/T, but results were non-significant. These results are comparable to a study conducted by Chi et al. (65), which demonstrated a significant improvement in clinical cure (OR = 1.62, 95% CI = 1.05–2.51) for C/T when compared to aminoglycosides/polymyxins or levofloxacin, while the difference in mortality between treatmentswas not statistically significant (65). It is important to note that our study focused on clinical outcomes and did not assess toxicity outcomes, such as acute kidney injury (AKI). However, other studies have found a higher incidence of AKI following treatment with the aminoglycoside/polymyxin group compared to the C/T (9, 66–72). The presence of AKI, regardless of its cause, leads to significant increases in morbidity, mortality, and healthcare-resource utilization. Additionally, AKI is increasingly recognized for causing long-term damage to various organ systems, including neuromuscular, cardiovascular, pulmonary, gastrointestinal, hepatobiliary, immunologic, hematologic, and metabolic systems (73). As such, the findings from this study should be considered a conservative estimate of the outcomes associated with C/T versus aminoglycoside/polymyxin-based therapies due to its design (73).

There were few comparator studies available to assess potential outcome differences between C/T and CZA. The results of the NMA did not provide conclusive evidence for either C/T or CZA being more favorable compared to the other for any outcome explored. More research is needed, particularly in patients with serious infections like pneumonia or bacteremia, as recent data suggest that C/T may offer some advantages over CZA for treating these infection types caused by MDR-*P. aeruginosa* (35). The results for clinical cure were not statistically significant, which is comparable to a study conducted in 45 patients with MDR-*P. aeruginosa* infections reporting improvement in clinical cure with C/T treatment versus CZA; however, statistical significance was not established (60% versus 48%, *P* = 0.432). Further, a retrospective study (CACTUS) conducted from 2016 to 2023 in 420 patients with MDR-*P. aeruginosa* pneumonia or bacteremia reported that there were no significant differences between C/T and CZA in terms of all-cause mortality, which aligns with our findings suggesting that C/T is non-inferior to CZA for all-cause mortality (35). This finding is similar in other resistance types as well; a prospective observational study evaluating patients infected with difficult-to-treat resistance *P. aeruginosa* found that 30-day mortality was similar between C/T-treated and CZA-treated patients (21% versus 17%; adjusted OR = 1.01) (74). Within our study, microbiological cure and LOS did not display statistically significant results (OR = 0.789, 95% CI = 0.303–1.842 and MD = 2.222, 95% CI = −3.759 to 5.914, respectively) for C/T treatment versus CZA, which is comparable to a study conducted by El Nekidy et al. (40) investigating C/T versus CZA for MDR-*P. aeruginosa* infections.

Additionally, outcomes that were not evaluated within our study due to limited data availability could aid in informing clinical decision-making by providing information around the superiority of C/T versus comparators. A comparative evaluation of non-COVID-19 adult hospitalized patients with MDR-*P. aeruginosa* pneumonia in the PINC AI Healthcare Database (2016–2022) suggested that C/T may offer some clinical benefits over CZA in terms of global patient outcomes (desirability of outcome ranking [DOOR] probability: 59.6%; 95% CI = 52.5%–66.8%), recurrence (7.9% versus 18.0%, *P* = 0.03), and 60-day pneumonia-related readmissions (11.1% versus 28.5%, *P* = 0.03).

The findings of our study have important implications for clinical practice (75). In the absence of head-to-head trials, real-world data may be used to inform clinical decision-making and policy-making (41, 76). There is often significant heterogeneity between real-world observational studies including variability in outcome definitions and patient populations, which limits the interpretation of results. In these situations, results from well-designed MA and NMA can be used to aid in rank ordering the outcomes associated with available treatments. Furthermore, the potential development of resistance to newer therapies must also be considered for clinical decision-making; in a small study (*n* = 21), resistance to C/T emerged in three patients treated for MDR-*P. aeruginosa* infections (55) and resistance to CZA has also been reported in case reports during treatment for carbapenem-resistant *Enterobacteriaceae* infections (77). Studies with larger sample sizes must be conducted for a more accurate and representative view of the clinical efficacy and healthcare-resource utilization effect of C/T versus CZA and aminoglycosides/polymyxins, as well as the resistance rates for these agents (35).

There were limited data available to compare C/T versus other antimicrobials, such as cefiderocol or imipenem-cilastatin-relebactam (IMI/REL); and therefore, publications reporting on these interventions were excluded during the feasibility assessment. As a result, no conclusions can be made regarding the relative effectiveness of these interventions versus C/T, as no comparisons were made. Further research should be conducted to investigate the relative effectiveness of these interventions to inform clinical decision-making and policy making.

This study was supported by Merck & Co., Inc. and two studies comparing C/T with C/A were also funded by Merck & Co., Inc. (38, 41). A key limitation of our study is the significant heterogeneity between observational studies, such as the definitions of outcomes and the time of outcome assessment, which were likely factors influencing the sizes of the CIs in the NMA for each outcome; this may have hindered any other potentially statistically significant results. The results of the MA and NMA could have also been affected by variation in treatment schedules, particularly as some treatments were grouped that likely had several differences in clinical practice, such as polymyxins and aminoglycosides. Variations or omissions of the assessment time of therapy in relation to culture collection day may also have impacted the results of the analyses. Additionally, the lack of data for other potential comparator treatments may have introduced bias as seen by the exclusion of most treatments included in the SLR PICOTS criteria for the MA and NMA. Furthermore, analyses comparing C/T and other treatments to provide a global assessment of patients’ experiences with MDR-*P. aeruginosa* infections were largely lacking. Further limitations of the NMA conducted include bias introduced by selective reporting of outcomes due to lack of data. In order to tackle the impact of these limitations, prioritization criteria were applied, and a feasibility assessment was conducted to remove any studies which would skew results, such as studies reporting sample sizes <10 and publications reporting mixed infection populations. Additionally, sensitivity analyses were conducted to adjust for differences in populations, study design, treatments, and outcomes as well as study quality and to test the sensitivity of NMA and MA results.

### Conclusions

In the absence of head-to-head trials, the real-world evidence indicates potential advantages of using C/T over older combination therapies for MDR-*P. aeruginosa* infections. Findings support C/T as a preferred treatment option with statistically significant improvements in clinical cure and all-cause mortality compared to aminoglycosides/polymyxins, but larger prospective studies with standardized outcome measures are needed to further inform clinical decision-making.

## MATERIALS AND METHODS

### Search strategies and selection criteria

The databases Embase, MEDLINE, EconLit, APA PsycInfo, and Evidence Based Medicine (EBM) Reviews were searched using the OVID platform to capture real-world effectiveness studies published from January 2009 to October 2024. Additional gray literature searches were conducted for key conferences: European Scientific Working Group on Influenza Conference, European Society of Paediatric Infectious Diseases, European Congress of Clinical Microbiology and Infectious Diseases, and IDWeek.

### Inclusion criteria

Titles and abstracts of retrieved citations, and subsequently full texts, were screened according to the pre-defined PICOTS eligibility criteria (Table S1) independently by two reviewers who were blinded to each other’s decisions. Relevant data from included publications were extracted into a concise DEF by one reviewer and verified by a second reviewer.

Prioritization criteria were applied to identify the final publications to be included in the SLR. To mitigate potential causes of heterogeneity, studies with low sample sizes (<10) such as case reports were removed. Additionally, only studies which exclusively reported on MDR-*P. aeruginosa* infections were included, as it was deemed inappropriate to compare MDR-*P. aeruginosa* with other bacterial infections. It was identified that the following outcomes—clinical cure, microbiological cure/failure, all-cause mortality, and LOS—were all widely reported upon; studies that did not report on these endpoints were excluded. PRISMA is outlined in Fig. 1. The included studies also underwent a quality assessment using the Joanna Briggs Institute risk of bias tool (Table S2).

### Feasibility assessment

A feasibility assessment was conducted, informed by the results of the SLR, to determine the viability and practicality of conducting quantitative synthesis of study results using meta-analytical or indirect treatment comparison techniques. For evidence synthesis, the feasibility assessment considered the 318 studies included in the SLR. The feasibility analysis excluded any publication that included comparators that were not aminoglycosides, polymyxins, or CZA due to a lack of comparator treatment data. To perform the evidence synthesis, some comparators were grouped by common treatment combinations (Table 3).

**TABLE 3:** Comparators by treatment grouping

Treatment group	Grouped treatments
C/T	C/T monotherapy C/T combination therapy
CZA	CZA monotherapy CZA combination therapy
Aminoglycosides/polymyxins	Aminoglycosides Polymyxins Combination therapies

### Data synthesis

#### Meta-analysis

To summarize the effectiveness of C/T in treating MDR-*P. aeruginosa* infections, an MA was conducted including C/T data from both single-arm and multi-arm trials to identify a single pooled estimate with a 95% CI across studies for all outcomes, allowing for one synthesized output to summarize multiple trials. If a study had missing outcome data for the relevant outcome, it was not included so as not to create bias. A proportional MA was performed for dichotomous outcomes (clinical cure, microbiological cure/failure, and all-cause mortality) (78, 79). This method of data synthesis allowed for the calculation of a pooled, overall proportion from a number of individual proportions, and the generation of a single summary estimate and its variance. For LOS, a continuous MA was conducted, as some of the studies were single arm, so it was not possible to adjust data through the standardized MD. R Project was used for the MA as the software is free, and the validated MA codes for different types of outcomes are publicly available and have been widely used in the past. R packages “meta” were used to perform the analyses.

#### Network meta-analysis

The NMA was performed in a Bayesian framework using Markov chain Monte Carlo techniques in the WinBUGS software version 1.4 to allow ranking of treatments in terms of efficacy. This method added robustness to the NMA as it used distributions in the absence of direct sampling. Heterogeneity between the studies was calculated using *I*^2^ statistic, with a higher score for the *I*^2^ statistic indicating higher levels of heterogeneity (80). This calculation provided the measure of variance not due to any sampling error, and this informed the choice of statistical model used (FE or RE model) after classification of low, moderate, or substantial heterogeneity. For binomial event data (clinical cure, microbiological cure/failure, and all-cause mortality), a binomial model with a logit link was used, and therefore, treatment effect versus C/T was measured using OR and 95% CI. For continuous data (LOS), a normal model with the identity link was used, and treatment effect versus C/T was measured using MD and 95% CIs. For each endpoint, two modeling approaches were conducted: an FE model which assumes that the relative effect of one treatment compared with another is the same across all trials containing these treatments (81), and an RE model that allows the treatment effects to differ while assuming these are all derived from a common distribution. The best-fitting model was determined using the DIC (82, 83); a higher DIC indicated a relative improvement in fit and the model with the highest DIC was used. Where the DIC values were similar between the FE and RE models, the RE model results are presented as this model can, to an extent, adjust for between-study heterogeneity. Where outcomes were missing uncertainty measures, it was necessary to impute values to enable the corresponding studies to be included in the analysis. Inputs required for dichotomous data are the number of events and sample size by treatment arm. For continuous data, the analysis required the mean and associated SE for a given treatment arm compared to the within-trial referent comparator.

#### Sensitivity analyses

To address potential sources of heterogeneity identified from the feasibility assessment, for all-cause mortality, clinical cure, and microbiological cure, sensitivity analyses were conducted to align on the definition of the endpoint considered and the timepoint of assessment as well as geographical location. The MA sensitivity analyses explored the effect of placing different confidence levels in the study data and evaluated the risk of bias in the results after considering the relative contribution of each source of evidence in the pooled estimates. For the NMA, the sensitivity analyses focused on excluding publication types that are likely to introduce bias or not provide full methods or patients' baseline summary data to allow for a full assessment of comparability. The sensitivity analyses excluded studies conducted in Asia across all clinical and healthcare-resource utilization outcomes and limited studies included based on outcomes at day 30.

## Data Availability

Data are available from the corresponding author upon request.

## SUPPLEMENTAL MATERIAL

Supplemental material aac.00735-25-s0001.docx Fig. S1; Table S1.

Table S2 aac.00735-25-s0002.xlsx Quality assessment of studies included in the meta-analysis and network meta-analysis.

ASM does not own the copyrights to Supplemental Material that may be linked to, or accessed through, an article. The authors have granted ASM a non-exclusive, world-wide license to publish the Supplemental Material files. Please contact the corresponding author directly for reuse.
