# Supplementary material for: A systematic literature review and (network) meta-analysis of the effectiveness of ceftolozane/tazobactam versus aminoglycosides/polymyxins and ceftazidime/avibactam for treating adult patients with multidrug-resistant Pseudomonas aeruginosa infections
Source: Antimicrob Agents Chemother. 2026 Jan 14;70(2):e00735-25. doi: 10.1128/aac.00735-25 (PMC12888884; doi:10.1128/aac.00735-25)
Supplement: Supplemental material — Fig. S1; Table S1. [file aac.00735-25-s0001.docx]

**Supplementary material**

**Figure S 1 - The forest plot for the network meta-analysis sensitivity analysis when restricting to studies (A) that reported results for clinical cure within 30 days, (B) that reported all-cause mortality at 30 days, (C) that reported microbiological cure relating to eradication by 30 days.**


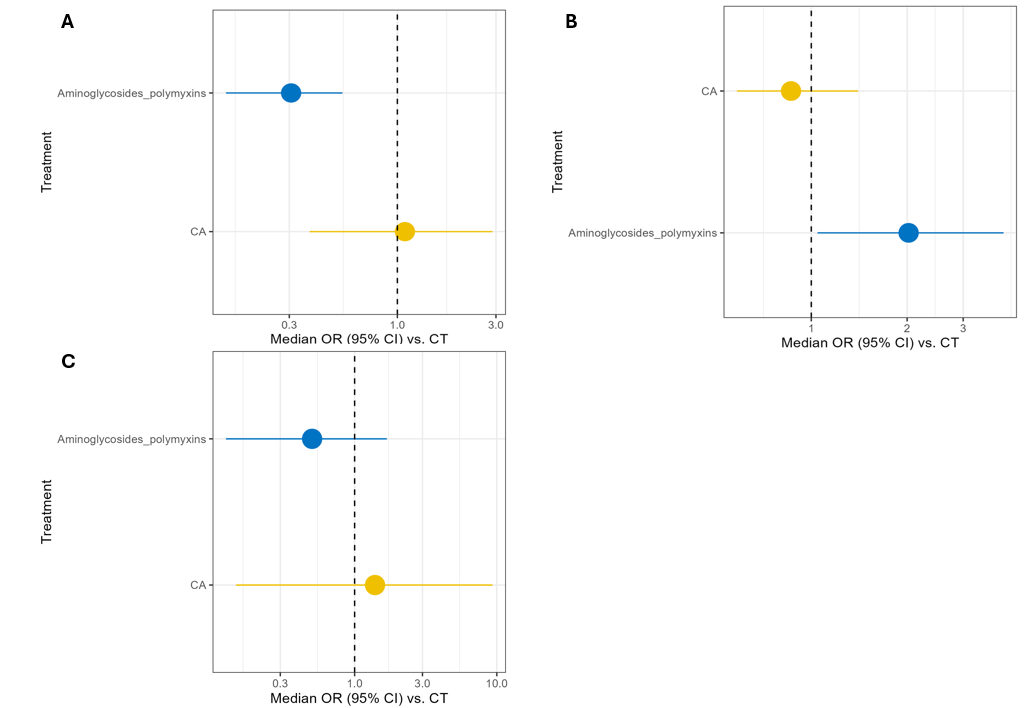


**Table S 1 - PICOTS criteria for inclusion/exclusion of identified publications for the systematic literature review**

|  | **Inclusion Criteria** | **Exclusion Criteria** |
| --- | --- | --- |
| **Population(s)** | Adult (aged ≥18 years) patients with MDR *P. aeruginosa* bacterial infections | Studies not reporting data on adult (aged ≥18 years) patients with *P. aeruginosa* bacterial infections |
| **Interventions** | Ceftolozane/tazobactam | Any interventions not listed in the inclusion criteria |
| **Comparators** | Piperacillin/tazobactam  Aztreonam  Ceftazidime/avibactam  Meropenem/vaborbactam  Cefiderocol  Imipenem/cilastatin/relebactam  Aminoglycoside  Polymyxins | Other treatments or therapies not listed in the inclusion criteria |
| **Outcomes** | Clinical outcomes: Clinical cure/failure, microbiological cure/failure, resistance development, all-cause in hospital mortality, 30-day mortality, In hospital infection-related mortality, reinfection, kidney-function outcomes (e.g.; changes in glomerular filtration rate, creatinine clearance, and acute kidney injury)  Economic outcomes: length of stay, admission to ICU, readmission, days of mechanical ventilation, receipt of mechanical ventilation, infection-related hospital cost/expenditure/expense, cost-effectiveness results, and budget impact results | Any outcome not in the inclusion criteria (e.g., PK/PD outcomes, susceptibility outcomes) |
| **Time** | Studies published from January 2009 to October 2024 | Studies published prior to January 2009* |
| **Study Design** | Non-randomized controlled clinical studies (e.g., non-interventional observational studies, cohort studies, case-control studies)  Economic evaluations (e.g., cost-effectiveness/BIM) | Randomized controlled studies  Non-clinical studies (e.g., *in vitro*)  Commentaries, editorials, literature reviews or letters without data presented  Abstracts and letters to the editor |
| **Other** | English language publications, human studies | Studies not published in English, non‑human studies |

BIM: Budget impact model; GN: Gram-negative; ICU: Intensive care unit; N/A: Not applicable; PD: Pharmacodynamics; PK: Pharmacokinetics; SAE: Serious adverse event.
